# Dr. Baillie

**Published:** 1823-10

**Authors:** 


### OBITUARY.

It is with feelings of no common regret, that we have to announce the death of Dr. BAILLIE, which took place at his seat in Gloucestershire, on the 23d ult. Dr. Baillie, alike distinguished as a physician, and amiable as a man, has run a career of honour and profit which falls to the lot of few. Acknowledged by the public and by his brethren as the undisputed head of the profession, he has left a blank which we can scarcely hope to see filled. In our next Number we shall present our readers with a short biographical sketch of this eminent individual,—the humble tribute of our respect for so much worth and talent.

